# Rosmarinic acid down‐regulates NO and PGE_2_ expression *via* MAPK pathway in rat chondrocytes

**DOI:** 10.1111/jcmm.13322

**Published:** 2017-09-25

**Authors:** We‐Ping Chen, Guo‐Jun Jin, Yan Xiong, Peng‐Fei Hu, Jia‐Peng Bao, Li‐Dong Wu

**Affiliations:** ^1^ Department of Orthopedic Surgery The Second Affiliated Hospital School of Medicine Zhejiang University Hangzhou China

**Keywords:** rosmarinic acid, osteoarthritis, matrix metalloproteinase, chondrocyte, interleukin‐1β, nitric oxide, prostaglandin E2

## Abstract

Rosmarinic acid (RosA) is a water‐soluble polyphenol, which can be isolated from many herbs such as orthosiphon diffuses and rosmarinus officinalis. Previous studies have shown that RosA possesses various biological properties. In this study, we investigate the anti‐osteoarthritic effects of RosA in rat articular chondrocytes. Chondrocytes were pre‐treated with RosA, followed by the stimulation of IL‐1β. Real‐time PCR and Western blot were performed to detect the expression of matrix metalloproteinase (MMP)‐1, MMP‐3 and MMP‐13. Nitric oxide and PGE_2_ production were measured by Griess reagent and enzyme‐linked immunosorbent assay (ELISA). The expression of mitogen‐activated protein kinase (MAPK) and nuclear factor‐κB (NF‐κB) was also investigated by Western blot analysis. We found that RosA down‐regulated the MMPs expression as well as nitric oxide and PGE_2_ production in IL‐1β‐induced chondrocytes. In addition, RosA inhibited p38 and JNK phosphorylation as well as p65 translocation. The results suggest that RosA may be considered a possible agent in the treatment of OA.

## Introduction

Osteoarthritis (OA) is an age‐related joint disorder, which was often seen in people over the age of 65 and lowers the life quality of the patients. The disease was characterized by the irreversible degradation of the articular cartilage. In normal cartilage, the chondrocytes, as the only cell in cartilage, maintain the balance between extracellular matrix (ECM) catabolism and anabolism 1. However, in OA, chondrocytes produce excessive catabolic factors such as matrix metalloproteinases (MMPs) and pro‐inflammatory cytokines, as a result, the balance was disturbed, leading to ECM degradation 2. MMPs were considered to play pivotal roles in cartilage degradation. These proteolytic enzymes can split collagen II and aggrecan, two main components of ECM, leading to cartilage degradation 3. In the light of the importance of MMPs in OA, inhibition of MMPs expression was considered as a strategy in the treatment of OA. Previous studies found that some MMPs inhibitors alleviate cartilage degradation in experimental OA 4, 5.

Recently, the role of inflammation in OA has been recognized. Inflammatory cytokines interleukin‐1‐beta (IL‐1β) and tumour necrosis factor (TNF)‐α play important roles in the progression of OA. These cytokines can induce NF‐κB and MAPK activation, leading to induction of inflammatory mediators including nitric oxide (NO) and prostaglandin E2 (PGE_2_). These inflammatory mediators may lead to ECM degradation and clinical manifestations 6.

Until now, non‐steroidal anti‐inflammatory drugs (NSAIDs) are still the main pharmacological treatment for OA, these drugs, with side‐effects in gastrointestinal and cardiovascular system, cannot stop or reverse OA progression 7. Therefore, the search for more effective and safer agents in the treatment of OA is still a challenge for researchers.

RosA is a water‐soluble polyphenol, which can be isolated from many herbs such as orthosiphon diffuses and rosmarinus officinalis. Previous studies have shown that RosA possesses various biological properties, including anti‐oxidative, anti‐inflammatory and anticancer properties 8, 9, 10. The role of RosA in rheumatoid arthritis was also reported 11, 12, 13. Moreover, Connelly *et al*. reported that daily consumption of the RosA improved stiffness and physical disability scores in adults with knee OA 14. However, the mechanism of RosA in OA is still unknown. In this study, we investigated the effects of RosA in chondrocytes. We found that RosA inhibited MMPs expression as well as the production of nitric oxide and PGE_2_ in chondrocytes.

## Materials and methods

### Reagents

RosA, dimethyl sulphoxide (DMSO), recombinant rat IL‐1β and MTT (3‐(4,5‐dimethyl‐thiazole‐2yl)‐2,5‐diphenyl tetrazolium bromide) were obtained from Sigma‐Aldrich (St. Louis, MO, USA). Antibodies against MMP‐1, MMP‐3, MMP‐13, inducible nitric oxide synthase(iNOS), cyclooxygenase‐2 (COX‐2), extracellular signal‐regulated kinase (ERK)1/2, p‐ERK1/2, p38, p‐p38, c‐Jun N‐terminal kinase (JNK), p‐JNK, β‐catenin p65 and β‐actin were purchased from Cell Signaling Technology (Beverly, MA, USA). Other reagents were obtained from Gibco BRL (Grand Island, NY, USA). RosA was dissolved in DMSO for stock preparation (100 mM).

### Cells culture and treatment

With the approval of Institutional Animal Care and Use Committee of Zhejiang University (Hangzhou, China), five two‐week‐old Sprague‐Dawley (SD) rats were used. Cartilage obtained from knee joints of rats for primary culture of chondrocytes as described previously 15. Cells were maintained in Dulbecco's modified eagle's medium (DMEM) supplemented with 10% FBS, 100 U/ml penicillin and 100 U/ml streptomycin. The medium was replaced every 2 days. Cells between passages 3 and 5 were used in the study. Cells were cultured with serum‐free medium overnight, treated with RosA (10, 50, 100 μM) for 1 hr and stimulated with IL‐1β (10 ng/ml) for another 24 hrs. Cells and the culture medium were collected for further study.

### Cells viability assay

Cells viability was measured using MTT assay. In brief, cells were seeded in 96‐well plates (5 × 10^3^/well), exposed to different concentrations of RosA. After 24‐hr incubation, cells were washed with PBS and incubated with 5 mg/ml MTT. The solution was removed and 150 μl DMSO was added. Cells viability was determined using the MTT assay.

### Nitric oxide and PGE_2_ measurement

The culture medium was collected for nitric oxide and PGE_2_ measurement. In brief, Griess reaction was used to assess the nitrite levels, and enzyme‐linked immunosorbent assay (ELISA) was used to measure PGE_2_ production according to the manufacturer's instructions (R&D Systems, Minneapolis, MN, USA). All assays were performed in duplicate.

### Quantitative real‐time polymerize chain reaction (PCR)

Total RNA was extracted from cells using Trizol reagent (Invitrogen, Carlsbad, CA, USA) according to the manufacture's protocol. Reverse‐transcribed to DNA templates using Malone Murine leukaemia virus reverse transcribe DNA synthesis kit (Promega, Madison, WI, USA) according to the manufacturer's instructions. Quantitative altimeter PCR was performed with IQ SYBR Green supermix PCR kit with the bicycle apparatus system (Bio‐Rad, Hercules, CA, USA). The sequence‐specific primers were as follows: for MMP‐1, forward: GCTTAGCCTTCCTTTGCTGTTGC; reverse: GACGTCTTCACCCAAGTTGTAGTAG, for MMP‐3, forward: CTGGGCTATCCGAGGTCATG; reverse: TGGACGGTTTCAGGGAGGC, for MMP‐13, forward: CAACCCTGTTTACCTACCCACTTAT; reverse: CTATGTCTGCCTTAGCTCCTGTC, for 18s rRNA, forward: GAATTCCCAGTAAGTGCGGGTCATA; reverse: CGAGGGCCTCACTAAACCATC. 18s rRNA was used as an endogenous reference gene. The relative eRNA expression was assessed using the 2^▵▵^CT method.

### Western blotting analysis

Cells were washed with ice‐cold phosphate‐buffered saline and harvested. Then, cells were treated with lysis buffer containing 50 mM Tris‐Cl, pH 7.4, 150 mM NaCl, 1 mM EDTA, 1 mM EGTA, 10 μg/ml aprotinin, 10 μg/ml leupeptin, 5 mM phenylmethylsulphonyl fluoride, 1 mM DTT and 1% Triton X‐100 for 30 min. on ice. The protein was extracted from cells, transferred to polyvinyl fluoride (PVDF) membranes, the membranes were incubated in Eris‐buffered saline Tween (TBS and 0.1% Tween 20) containing 5% bovine serum albumin overnight at 4°C, followed by primary antibodies (MMP‐1, MMP‐3, MMP‐13, Enos, COX‐2, ERK1/2, p‐ERK1/2, p38, p‐p38, JNK, p‐JNK, β‐catenin, p65 and β‐actin). After washing, membranes were incubated for 1 hr at room temperature with HAP‐linked secondary antibodies. Finally, the filter was incubated with the electroencephalographic substrate and exposed to X‐ray film (Kodak, Hangzhou, China).

### Statistical analysis

Data are expressed as means ± standard deviation. Data were analysed using a one‐way anova test. Dunnett‐t method was used as the post‐test in the anova. A value of *P* < 0.05 was taken to indicate statistical significance.

## Results

### Effects of RosA on cells viability

**Figure 1:**
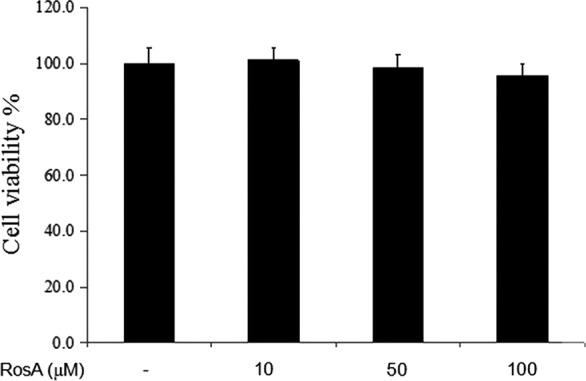
Effects of RosA on cell viability. Cells were seeded in 96‐well plates and cultured with different concentrations of RosA for 24 hrs, followed by MTT assay analysis. Data presented are the means ± standard deviation (SD) of three independent experiments.

Cells were exposed to RosA for 24 hrs, and the results showed that RosA exerted no adverse effects on cell viability (Fig. 1).

### Effects of RosA on nitric oxide and PGE_2_ production

**Figure 2:**
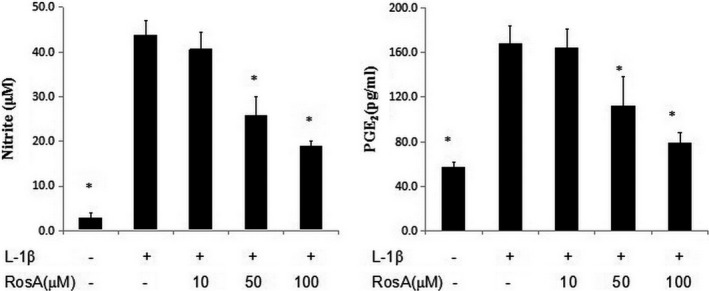
Effects of RosA on nitric oxide and PGE_2_ production in chondrocytes. Chondrocytes were pre‐treated with various concentrations of RosA for 1 hrs prior to IL‐1β (10 ng/ml) for 24 hr. Conditioned media were collected for nitrite and PGE_2_ measurement. Data are expressed as mean ± standard deviation (SD). *P < 0.05 compared with cells stimulated with IL‐1β only. The experiment is representative of three experiments performed.

**Figure 3:**
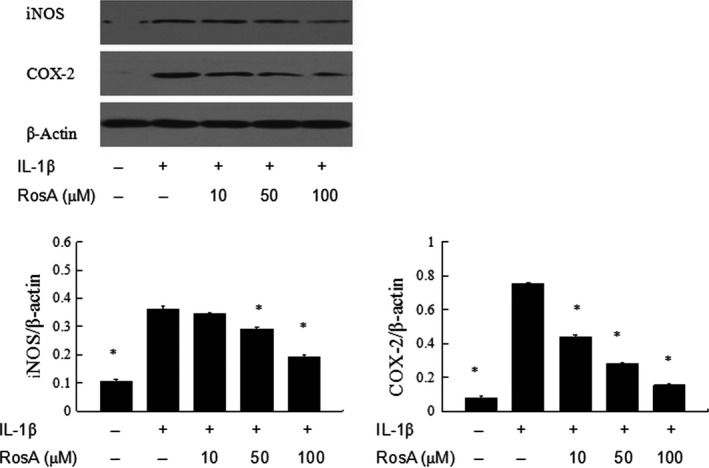
Effects of RosA on iNOS and COX‐2 expression in chondrocytes. Chondrocytes were pre‐treated with various concentrations of RosA for 1 hr prior to IL‐1β (10 ng/ml) for 24 hr. The protein levels of iNOS and COX‐2 in chondrocytes were assessed by Western blot analysis. The experiment is representative of three experiments performed. Data are expressed as mean ± standard deviation (SD). *P < 0.05 compared with cells stimulated with IL‐1β only.

The production of nitric oxide was assessed by Griess reaction, and the production of PGE_2_ was detected by ELISA. Treatment with RosA resulted in significantly down‐regulation of nitric oxide and PGE_2_ production induced by IL‐1β in chondrocytes (Fig. 2). In addition, RosA also inhibited the protein expression of iNOS and COX‐2 in chondrocytes (Fig. 3).

### Effects of RosA on MMPs expression in chondrocytes

**Figure 4:**
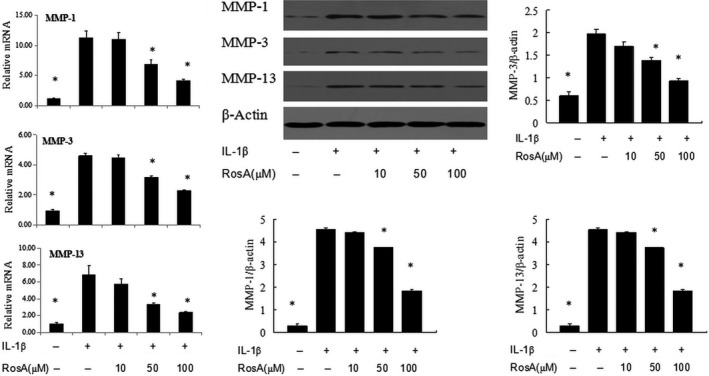
Effect of RosA on MMP‐1, MMP‐3 and MMP‐13 expression in chondrocytes. Cells were treated with RosA for 1 hr prior to treatment with IL‐1β (10 ng/ml) and were collected after 24 hrs. Quantitative real‐time PCR and Western blot analyses were performed to analyse MMP‐1, MMP‐3 and MMP‐13 mRNA and protein expression, respectively. Data are expressed as mean ± standard deviation (SD). *P < 0.05 compared with cells stimulated with IL‐1β only.

The effects of RosA on the gene expression of MMP‐1, MMP‐3 and MMP‐13 in IL‐1β‐induced chondrocytes were assessed by PCR. RosA significantly inhibited the IL‐1β‐induced gene expression of MMP‐1, MMP‐3 and MMP‐13 in a dose‐dependent manner. RosA also reduced the protein expressions of MMP‐1, MMP‐3 and MMP‐13 in chondrocytes (Fig. 4).

### Effects of RosA on MAPK activation in chondrocytes

**Figure 5:**
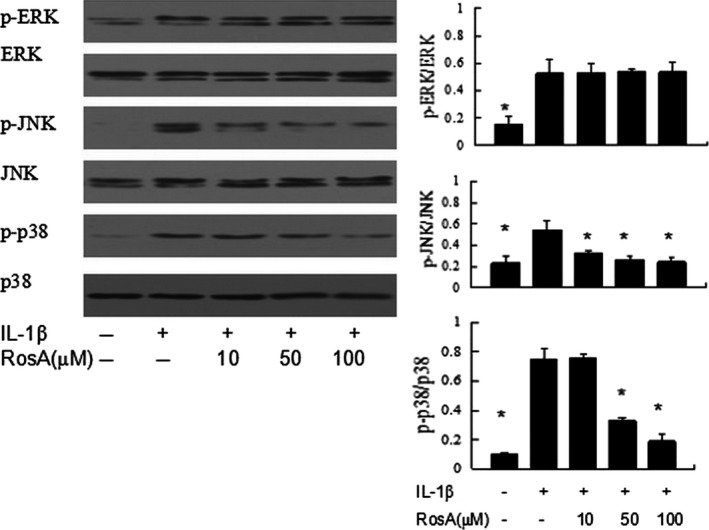
Effect of RosA on IL‐1β‐induced phosphorylation of MAPKs in chondrocytes. Cells were pre‐incubated with various concentrations of RosA for 2 hrs, followed by stimulation with IL‐1β (10 ng/ml) for 30 min. Phosphorylation of extracellular signal‐regulated kinase (ERK), p38 and c‐jun amino‐terminal kinase (JNK) was assessed by Western blot. Data are expressed as mean ± standard deviation (SD). *P < 0.05 compared with cells stimulated with IL‐1β only.

MAPK are essential signalling pathway in OA pathophysiology. Previous studies showed that RosA can affect the activation of MAPK. Thus, in this study, we investigated the effects of RosA on MAPK in IL‐1β‐stimulated chondrocytes. Phosphorylation of ERK1/2, p38 and JNK was increased by IL‐1β, and RosA inhibited the phosphorylation of JNK and p38 whereas ERK was unaffected (Fig. 5).

### Effects of RosA on NF‐κB activation

**Figure 6:**
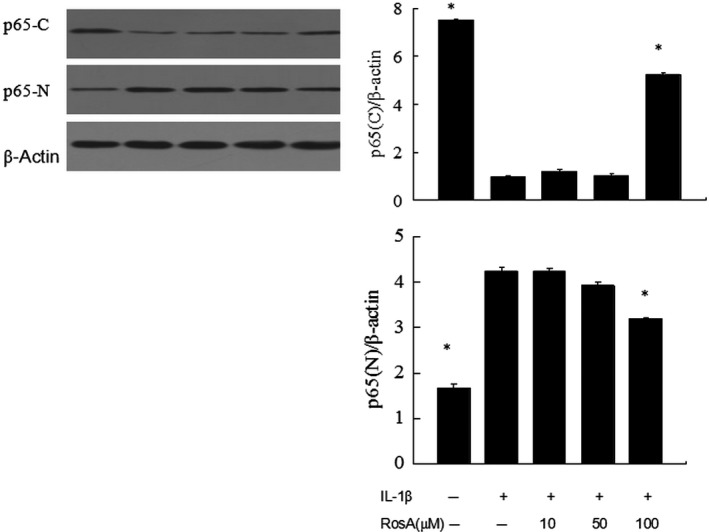
Effects of RosA on NF‐κB activation. Cells were pre‐incubated with various concentrations of RosA with or without IL‐1β. Western blot was performed to analyse the expression of p65 in cytoplasm and nucleus .The results showed that RosA inhibited IL‐1β‐stimulated translocation of p65 from the cytoplasm to the nucleus in chondrocytes.

It has been considered that activation of NF‐κB signalling pathway plays an important role in OA; as p65 translocation is an indicator of NF‐κB activation by IL‐1β stimulation, we checked the protein levels of p65 in the nuclear and cytoplasmic. The results showed that IL‐1β stimulation resulted in significant translocation of p65 from the cytoplasm to the nucleus (Fig. 6).

### Effects of RosA on Wnt/β‐catenin signalling pathway

**Figure 7:**
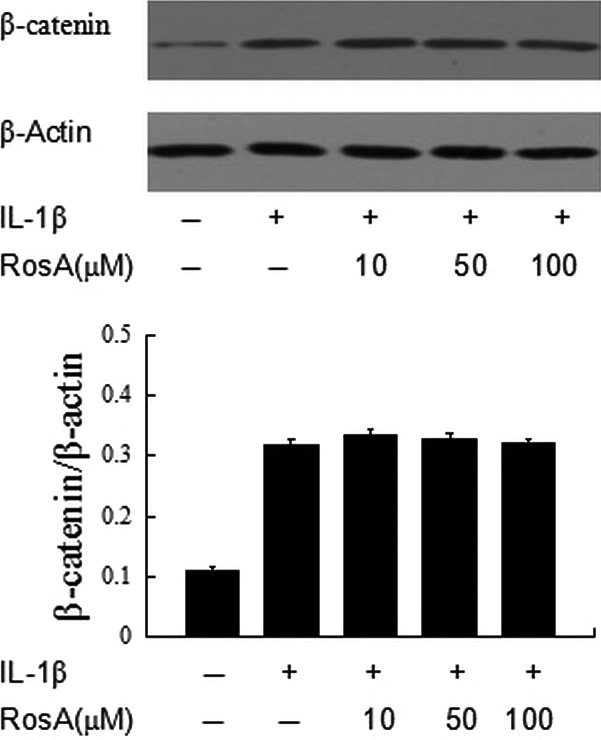
Effects of RosA on β‐catenin activation. Cells were pre‐incubated with various concentrations of RosA with or without IL‐1β. Western blot was performed to analyse the expression of β‐catenin. Data are expressed as mean ± standard deviation (SD). *P < 0.05 compared with cells stimulated with IL‐1β only.

We also detected the effects of RosA on Wnt/β‐catenin signalling pathway because it was closely related to MMPs expression in OA. We found that stimulation with IL‐1β lead to increase in β‐catenin expression, but RosA exerted no effects on β‐catenin activation (Fig. 7).

## Discussion

Osteoarthritis is a widespread joint disease, which is characterized by degradation of articular cartilage. Accumulating studies showed that some natural products may be the potential agents for the treatment of OA 16. In the present study, we evaluated the anti‐osteoarthritic effects of RosA in chondrocytes, and we found that RosA significantly decreased the IL‐1β‐induced gene and protein expression of MMP‐1, MMP‐3 and MMP‐13 in chondrocytes. RosA also inhibited the IL‐1β‐induced expression of iNOS and COX‐2 as well as the production of nitric oxide and PGE_2_. Furthermore, RosA inhibited the IL‐1β‐induced MAPK and NF‐κB activation.

Cartilage degradation is the hallmark feature of OA, thus inhibiting the degradation of cartilage is an important issue for the treatment of OA. Aggrecan and type II collagen are known to be the most components of cartilage matrix. It is well known that MMPs are the main matrix catabolic enzymes leading to ECM degradation and subsequent cartilage degradation in OA. Previous study showed that MMP‐13 is a primary collagenase, which is responsible for type II collagen degradation in OA 17. In addition, MMP‐1 and MMP‐3 are also involved in ECM degradation. In the present study, it was demonstrated that RosA significantly inhibited the IL‐1β‐induced expression of MMP‐1, MMP‐3 and MMP‐13 in chondrocytes. Therefore, inhibiting the activities of MMPs may be one of the mechanisms by which RosA prevents cartilage degradation.

It is well established that inflammation is associated with the progression of OA 18. IL‐1β, as a major inflammatory cytokine, plays an important role in the progression of OA. Stimulation with IL‐1β could result in up‐regulation of MMPs, aggrecanases and other catabolic enzymes in OA 19. In addition, IL‐1β can up‐regulate the expression of iNOS and COX‐2 as well as the production of nitric oxide and PGE_2_ in OA. In the present study, we used IL‐1β to mimic the environment of OA *in vitro*, the results were similar to previous study showed that IL‐1β induced the expression of MMP‐1, MMP‐3 and MMP‐13 as well as the production of nitric oxide and PGE_2_ in chondrocytes 20.

Nitric oxide and PGE_2_ are inflammatory mediators, which were implicated in the degradation of articular cartilage *via* inhibiting the synthesis of ECM; thus, inhibition of nitric oxide or PGE_2_ can attenuate the progression of OA 21. In the present study, we demonstrated that RosA inhibited IL‐1β‐induced nitric oxide and PGE_2_ production as well as iNOS and COX‐2 expression in chondrocytes. Previous study showed that RosA inhibited the production of nitric oxide and PGE_2_ in RAW 264.7 mouse macrophages 22. Taken together, we have been suggested that RosA may exert beneficial effects in OA *via* inhibiting inflammatory progression.

NF‐κB signalling pathway has been shown to be involved in inflammatory progression. Previous study showed that NF‐κB is an important regulator in the induction of iNOS and COX‐2 in chondrocytes 23, 24. In the present study, we investigated the effects of RosA on NF‐κB signalling pathway. We found that NF‐κB p65 translocated from the cytoplasm to the nucleus after IL‐1β stimulation, and RosA blocked the nuclear translocation of the p65. Our findings are consistent with other studies, which found that RosA can inhibit NF‐κB activation 25, 26.

We investigated the MAPK signalling pathway because MAPKs, including ERK, p38 and JNK, play important roles in the regulation of MMPs expression in OA 27. Thus, inhibition of MAPK is a strategy in the treatment of OA. Some agents inhibited MMPs expression *via* suppressing MAPK activation in chondrocytes 28, 29. In the present study, we demonstrated that RosA inhibited IL‐1β‐induced phosphorylation of JNK and p38 while ERK was not affected. Our results were partly supported by previous studies, for example, Kim *et al*. found that RosA inhibited p38, ERK and JNK activation in bone marrow‐derived dendritic cell 30. EI Omri *et al*. found that RosA activated cholinergic activities in PC12 cells through inhibiting phosphorylation of ERK1/2 31. These findings indicate that RosA exerted pharmacologic properties with respect to the inhibition of MAPK signalling pathway. The disparity may be attributable to differences in RosA exposure time or cell type.

In conclusion, our results demonstrated that RosA inhibited MMPs expression as well as nitric oxide and PGE_2_ production in chondrocytes through the modulation of NF‐κB and MAPK signalling pathways. These findings indicate a potential therapeutic role for RosA in OA.

## Conflict of interests

The authors declare that they have no conflict of interests.

## References

[1] Goldring MB . The role of the chondrocyte in osteoarthritis. Arthritis Rheum. 2000; 43: 1916–26. 11014341 10.1002/1529-0131(200009)43:9<1916::AID-ANR2>3.0.CO;2-I

[2] Maldonado M , Nam J . The role of changes in extracellular matrix of cartilage in the presence of inflammation on the pathology of osteoarthritis. Biomed Res Int. 2013; 2013: 1–10. http://dx.doi.org/10.1155/2013/284873 10.1155/2013/284873 PMC3771246 24069595

[3] Smith GN Jr . The role of collagenolytic matrix metalloproteinases in the loss of articular cartilage in osteoarthritis. Front Biosci. 2006; 11: 3081–95. 16720377 10.2741/2034

[4] Li NG , Shi ZH , Tang YP , *et al* New hope for the treatment of osteoarthritis through selective inhibition of MMP‐13. Curr Med Chem. 2011; 18: 977–1001. 21254976 10.2174/092986711794940905

[5] Burrage PS , Brinckerhoff CE . Molecular targets in osteoarthritis: metalloproteinases and their inhibitors. Curr Drug Targets. 2007; 8: 293–303. 17305507 10.2174/138945007779940098

[6] Daghestani HN , Kraus VB . Inflammatory biomarkers in osteoarthritis. Osteoarthritis Cartilage. 2015; 23: 1890–6. 26521734 10.1016/j.joca.2015.02.009 PMC4630669

[7] Hungin AP , Kean WF . Nonsteroidal anti‐inflammatory drugs: overused or underused in osteoarthritis? Am J Med. 2001; 110: 8S–11S. 11165988 10.1016/s0002-9343(00)00628-8

[8] Rocha J , Eduardo‐Figueira M , Barateiro A , *et al* Anti‐inflammatory effect of rosmarinic acid and an extract of Rosmarinus officinalis in rat models of local and systemic inflammation. Basic Clin Pharmacol Toxicol. 2015; 116: 398–413. 25287116 10.1111/bcpt.12335

[9] Wu CF , Karioti A , Rohr D , *et al* Production of rosmarinic acid and salvianolic acid B from callus culture of Salvia miltiorrhiza with cytotoxicity towards acute lymphoblastic leukemia cells. Food Chem. 2016; 201: 292–7. 26868579 10.1016/j.foodchem.2016.01.054

[10] Corral‐Lugo A , Daddaoua A , Ortega A , *et al* Rosmarinic acid is a homoserine lactone mimic produced by plants that activates a bacterial quorum‐sensing regulator. Sci. Signal. 2016; 9(409): ral. 10.1126/scisignal.aaa8271 26732761

[11] Pearson W , Fletcher RS , Kott LS . Oral rosmarinic acid‐enhanced Mentha spicata modulates synovial fluid biomarkers of inflammation in horses challenged with intra‐articular LPS. J Vet Pharmacol Ther. 2012; 35: 495–502. 22070392 10.1111/j.1365-2885.2011.01343.x

[12] Youn J , Lee KH , Won J , *et al* Beneficial effects of rosmarinic acid on suppression of collagen induced arthritis. J Rheumatol. 2003; 30: 1203–7. 12784390

[13] Hur YG , Suh CH , Kim S , *et al* Rosmarinic acid induces apoptosis of activated T cells from rheumatoid arthritis patients *via* mitochondrial pathway. J Clin Immunol. 2007; 27: 36–45. 17195044 10.1007/s10875-006-9057-8

[14] Connelly AE , Tucker AJ , Tulk H , *et al* High‐rosmarinic acid spearmint tea in the management of knee osteoarthritis symptoms. J Med Food. 2014; 17: 1361–7. 25058311 10.1089/jmf.2013.0189 PMC4259186

[15] Chen WP , Hu PF , Bao JP , *et al* Morin exerts antiosteoarthritic properties: an *in vitro* and *in vivo* study. Exp Biol Med. 2012; 237: 380–6. 10.1258/ebm.2011.011271 22496430

[16] Cameron M , Chrubasik S . Topical herbal therapies for treating osteoarthritis. Cochrane Database Syst Rev. 2013; 5: CD010538. 10.1002/14651858.CD010538 PMC4105203 23728701

[17] Troeberg L , Nagase H . Proteases involved in cartilage matrix degradation in osteoarthritis. Biochim Biophys Acta. 2012; 1824: 133–45. 21777704 10.1016/j.bbapap.2011.06.020 PMC3219800

[18] Liu‐Bryan R , Terkeltaub R . Emerging regulators of the inflammatory process in osteoarthritis. Nat Rev Rheumatol. 2015; 11: 35–44. 25266449 10.1038/nrrheum.2014.162 PMC4374654

[19] Santangelo KS , Nuovo GJ , Bertone AL . *In vivo* reduction or blockade of interleukin‐1beta in primary osteoarthritis influences expression of mediators implicated in pathogenesis. Osteoarthritis Cartilage. 2012; 20: 1610–8. 22935786 10.1016/j.joca.2012.08.011 PMC3478416

[20] Abella V , Scotece M , Conde J , *et al* The novel adipokine progranulin counteracts IL‐1 and TLR4‐driven inflammatory response in human and murine chondrocytes *via* TNFR1. Sci Rep. 2016; 6: 1–9. 26853108 10.1038/srep20356 PMC4745010

[21] Li N , Rivéra‐Bermúdez MA , Zhang M , *et al* LXR modulation blocks prostaglandin E2 production and matrix degradation in cartilage and alleviates pain in a rat osteoarthritis model. Proc Natl Acad Sci USA. 2010; 107: 3734–9. 20133709 10.1073/pnas.0911377107 PMC2840473

[22] Huang N , Hauck C , Yum MY , *et al* Rosmarinic acid in Prunella vulgaris ethanol extract inhibits lipopolysaccharide‐induced prostaglandin E2 and nitric oxide in RAW 264.7 mouse macrophages. J Agric Food Chem. 2009; 57: 10579–89. 19919113 10.1021/jf9023728 PMC2795400

[23] Marcu KB , Otero M , Olivotto E , *et al* NF‐kappaB signaling: multiple angles to target OA. Curr Drug Targets. 2010; 11: 599–613. 20199390 10.2174/138945010791011938 PMC3076145

[24] Rigoglou S , Papavassiliou AG . The NF‐kappaB signalling pathway in osteoarthritis. Int J Biochem Cell Biol. 2013; 45: 2580–4. 24004831 10.1016/j.biocel.2013.08.018

[25] Moon DO , Kim MO , Lee JD , *et al* Rosmarinic acid sensitizes cell death through suppression of TNF‐alpha‐induced NF‐kappaB activation and ROS generation in human leukemia U937 cells. Cancer Lett. 2010; 288: 183–91. 19619938 10.1016/j.canlet.2009.06.033

[26] Jeong HJ , Choi Y , Kim MH , *et al* Rosmarinic acid, active component of Dansam‐Eum attenuates ototoxicity of cochlear hair cells through blockage of caspase‐1 activity. PLoS One. 2011; 6: e18815. 21526214 10.1371/journal.pone.0018815 PMC3078149

[27] Sondergaard BC , Schultz N , Madsen SH , *et al* MAPKs are essential upstream signaling pathways in proteolytic cartilage degradation–divergence in pathways leading to aggrecanase and MMP‐mediated articular cartilage degradation. Osteoarthritis Cartilage. 2010; 18: 279–88. 19932675 10.1016/j.joca.2009.11.005

[28] Davidson RK , Jupp O , de Ferrars R , *et al* Sulforaphane represses matrix‐degrading proteases and protects cartilage from destruction *in vitro* and *in vivo* . Arthritis Rheum. 2013; 65: 3130–40. 23983046 10.1002/art.38133 PMC4240673

[29] Hamamura K , Lin CC , Yokota H . Salubrinal reduces expression and activity of MMP13 in chondrocytes. Osteoarthritis Cartilage. 2013; 21: 764–772. 23473976 10.1016/j.joca.2013.02.657

[30] Kim HK , Lee JJ , Lee JS , *et al* Rosmarinic acid down‐regulates the LPS‐induced production of monocyte chemoattractant protein‐1 (MCP‐1) and macrophage inflammatory protein‐1alpha (MIP‐1alpha) *via* the MAPK pathway in bone‐marrow derived dendritic cells. Mol Cells. 2008; 26: 583–9. 18799930

[31] El Omri A , Han J , Yamada P , *et al* Rosmarinus officinalis polyphenols activate cholinergic activities in PC12 cells through phosphorylation of ERK1/2. J Ethnopharmacol. 2010; 131: 451–8. 20633629 10.1016/j.jep.2010.07.006

